# Proposing a hybrid technique of feature fusion and convolutional neural network for melanoma skin cancer detection

**DOI:** 10.1016/j.jpi.2023.100341

**Published:** 2023-10-13

**Authors:** Md. Mahbubur Rahman, Mostofa Kamal Nasir, Md. Nur-A-Alam, Md. Saikat Islam Khan

**Affiliations:** a Department of Computer Science and Engineering, Bangladesh University of Business and Technology, Mirpur-2, Dhaka 1216, Bangladesh; b Department of Computer Science and Engineering, Mawlana Bhashani Science and Technology University, Tangail, Bangladesh; c Department of CSE, Dhaka International University, Dhaka 1205, Bangladesh

**Keywords:** Convolutional Neural Network (CNN), Histogram-Oriented Gradient (HOG), Local Binary Pattern (LBP), Speed Up Robust Feature (SURF), Hybrid Feature Extractor (HFE), Fast Bounding Box (FBB), Modified Anisotropic Diffusion Filtering (MADF)

## Abstract

Skin cancer is among the most common cancer types worldwide. Automatic identification of skin cancer is complicated because of the poor contrast and apparent resemblance between skin and lesions. The rate of human death can be significantly reduced if melanoma skin cancer could be detected quickly using dermoscopy images. This research uses an anisotropic diffusion filtering method on dermoscopy images to remove multiplicative speckle noise. To do this, the fast-bounding box (FBB) method is applied here to segment the skin cancer region. We also employ 2 feature extractors to represent images. The first one is the Hybrid Feature Extractor (HFE), and second one is the convolutional neural network VGG19-based CNN. The HFE combines 3 feature extraction approaches namely, Histogram-Oriented Gradient (HOG), Local Binary Pattern (LBP), and Speed Up Robust Feature (SURF) into a single fused feature vector. The CNN method is also used to extract additional features from test and training datasets. This 2-feature vector is then fused to design the classification model. The proposed method is then employed on 2 datasets namely, ISIC 2017 and the academic torrents dataset. Our proposed method achieves 99.85%, 91.65%, and 95.70% in terms of accuracy, sensitivity, and specificity, respectively, making it more successful than previously proposed machine learning algorithms.

## Highlights

• A modified filtering method is used to remove multiplicative speckle noise.
• A hybrid fused vector of HOG, LBP, SURF, and CNN is used to find optimum features.
• An entropy-based feature selection method is used to select meaningful features.
• VGG19 technique with hybrid feature fusion outperforms prior studies for skin cancer.

## Introduction

Human skin is the largest organ that acts as the cover of the body.^1^ Skin cancer appears when a cell of the skin grows abnormally, such as a hard red nodule or a scaly growth that produces a crust or a sore that does not heal.^2^ So, lighter skin, sunburns, or family history can increase the risk of skin cancer.^3^ UV lights from the sun can damage the unprotected skin’s DNA and alter the DNA. Thus, uncontrolled cell growth leads to cancer.^4^ Squamous cell carcinoma, melanoma, and basal cell carcinoma mutations may begin to alter the derma, resulting in skin cancer. Skin cancer shows such an alarming rate. According to WHO, currently there are more than 2 million cases of non-melanoma skin cancers and around 130 000 people are affected every year around the world.^5^^,^^6^ If melanoma cancer spreads, it will be deadly, while, it is curable in its early stages. Out of 5 stages, for melanoma stages 0, 1, and 2 has 98.4% survival rate. The survival rate at stages 3 and 4 are 63.6% and 22.4%, respectively.^7^^,^^8^ Hence, if we can detect melanoma skin cancer quickly, we can reduce human mortality rate with some initial treatment. Currently, detection and level-based classification are mostly done by doctors manually, which are prone to human error. Machine learning approaches can help to increase detection speed and accuracy.^9^

Here, we propose a new machine learning approach to classify melanoma and non-melanoma skin cancer from dermatology images. To build this model, we first truncate the selected Region of Interest (ROI) to erase the noisy and undesired parts and after that convert images RGB to grayscale. Furthermore, we propose 2 feature extractors namely, Hybrid Feature Extractor (HFE), and Convolutional Neural Network (CNN)-based feature extractor. The HFE combines 3 feature extractors namely, Histogram-Oriented Gradient (HOG), Local Binary Pattern (LBP), and Speed Up Robust Feature (SURF) and generate 1 fused feature vector. Proposed technique selects optimal features from non-optimized fused features utilizing an entropy-based feature selection mechanism. In the next step, the CNN is utilized to extract additional features. These 2-feature vectors are then combined to train build our classification model.

Input dermatology images is speckle attacked and low-quality. Unlike prior studies that employ classic filtering approaches, we suggest a modified form of anisotropic diffusion filtering to enhance the prediction performance. Apostrophic diffusion filtering method removes multiplicative noise present from the train and test images. In this way, it can efficiently overcome the image quality constraint. The test images are then subjected to the feature extraction approach. Finally, the classifier determines whether or not dermatology images are melanoma or non-melanoma. Furthermore, most of prior studies have utilized limited data to diagnose skin cancer. Hence, it is hard to generalize those models. To address this shortcoming, here we employ 2 balanced genuine datasets and merged them to have a larger benchmark.

The key contribution of this research is:

• A modified anisotropic diffusion filtering method is proposed to remove multiplicative speckle noise from dermoscopy images.
• In order to discover the optimum features, a hybrid fused vector is suggested that combines HOG, LBP, SURF, and CNN.
• An entropy-based feature selection method is used to select meaningful features from fused features vector.
• A CNN-based VGG19 technique with hybrid feature fusion is proposed that outperforms prior studies for skin cancer detection found in the literature.

## Related work

The medical and biological datasets are growing in size very rapidly. To analyze such big and complex data, artificial intelligence and machine learning algorithms have become popular.^10^, ^11^, ^12^, ^13^, ^14^, ^15^, ^16^, ^17^ In particular, machine learning and deep learning techniques have been widely used to analyze imaging data.^15^^,^^18^, ^19^, ^20^, ^21^, ^22^, ^23^, ^24^, ^25^, ^26^ Many researchers have used different techniques in melanoma skin cancer detection including dermatology image of skin. Using a deep neural network to detect and classify skin cancer is a challenging task. A large number of researchers have used different techniques for melanoma skin cancer detection.

In several early studies, Jain and Pise, Patel et al., and Rejeesh^27^, ^28^, ^29^ introduced a novel system to detect melanoma skin cancer. They preprocessed skin lesion input image to get the high-quality image. They also used thresholding and edge detection techniques for segmentation purpose. Then, they extracted features from segmented image by using geometry-based features and ABCD (Asymmetry, Border, Color, and Diameter) features. These extracted features classified the image as ordinary skin and melanoma skin cancer. Later on, Alquran et al.^30^ employed Support Vector Machine (SVM) to detect normal skin and abnormal skin. To build this model, first they preprocessed the dermoscopy image using threshold value. The gray level co-occurrence matrix was then utilized to extract features, and feature selection was done using Principal Component Analysis (PCA) approaches. For classification purpose, they used support vector machine and calculated total dermoscopy score.

Li et al.^31^ proposed 2 deep learning models for lesion region detection, dermoscopic feature extraction, and classification. For segmentation and classification, they utilized a fully connected Convolutional Residual Networks (FCRN). They also used Lesion Index Calculation Unit (LICU) approach to refine the classification outcome. Finally, they used CNN for dermoscopic feature extraction. The highest given prediction accuracy using their framework is 91.2%. In a different study, Vijayalakshmi et al.^32^ assessed the problem using 3 phases such as: data collection and augmentation, model design, and prediction. They used CNN and SVM algorithms, and augmented it with different image processing tools and reported 85% prediction accuracy. Later on, Pramanik and Chakraborty^33^ analyzed current technology to detect skin cancer. In their research, K-Means clustering is used for image segmentation, Wavelet Transform for feature extraction, and SVM.

In a different study, Esteva et al.^34^ proposed a method for skin cancer categorization using a pretrained Inception V3 CNN model. They analyzed 129 450 clinical skin cancer images and 3374 dermoscopic images and reported classification accuracy of 72%. Later on, Jayapriya et al.^35^ suggested a deep convolutional neural network-based layout to tackle this problem. On the ISBI challenge dataset, they built a Fully Convolutional Residual Network (FCRN) for real skin coup segmentation and produced 50 layers for the classification of melanoma cancer, with the best classification result of 88.92%. Using Google's V4 CNN model, Haenssle et al.^36^ employed deep convolutional neural networks to identify skin cancer from dermoscopy images in 2018 and achieved 86.6% in terms of specificity. More recently, Dorj et al.^37^ proposed a hybrid model to classify skin lesion cancer using AlexNet convolutional neural network and ECOC SVM methods. Pre-trained AlexNet used for extracting features and ECOC SVM was used as a classifier. They reported 95.1% in terms of maximum value of average accuracy. Most recently, Sies et al.^38^ studied the possible correlation between gender and skin cancer. In their study, 40% of the data for female patients and 60% of the data for male patients were used. Finally, using authorized CNN model, they reported that there is no gender bias with respect to this disease.

After reviewing the literature, it was discovered that many machine learning classifiers, including CNN, SVM, K-Means, and others, produced inconsistent results. However, each of these methods and outcomes yields a distinct set of findings, which is rather perplexing. Skin cancer is more harmful in stages 3 and 4 than in previous stages. We must forecast it earlier and more precisely. As a result, motivation is gained to deal with skin cancer in order to use hybrid methods to produce a more precise outcome. Because of this, a simple machine learning strategy based on feature fusion is used in this study, which has the highest accuracy compared to earlier research. The related works are summarized in Table 1.

**Table 1:** Summary of recent studies on skin cancer.

Dataset	Classifier and training algorithm	Description	Results (%)
ISIC 2017 dataset^31^	FCRN, LICU, CNN	Lesion segmentation using FCRN, dermoscopic feature extraction using CNN, and lesion classification using LICU	91.20
ISIC dataset^32^	CNN, SVM	Data augmentation using CNN and prediction using SVM	85.00
Kaggle dataset^33^	K-Means, SVM	K-Means clustering for segmentation, wavelet transform for feature extraction, and SVM for classification	87.58
Standard dataset^34^	CNN	Only pixels and disease labels were used as input data and features are extracted by Inception V4 model	72.10
ISIC 2017 dataset^35^	CNN+FCRN	VGG-16 and Google Net are used in segmentation and classified with CNN	88.92
Standard dataset^36^	Deep CNN+Google Inception V4	Data analyzed in 2 consecutive levels and Inception V4 model used for feature extraction	86.60
ISIC dataset^37^	ECOC SVM+CNN	AlexNet used in extracting features and ECOC SVM used as classifier	95.10
Standard dataset^38^	CNN	Investigated with CNN for sex-related biasness	98.40

## Research methodology

**Fig. 1:**
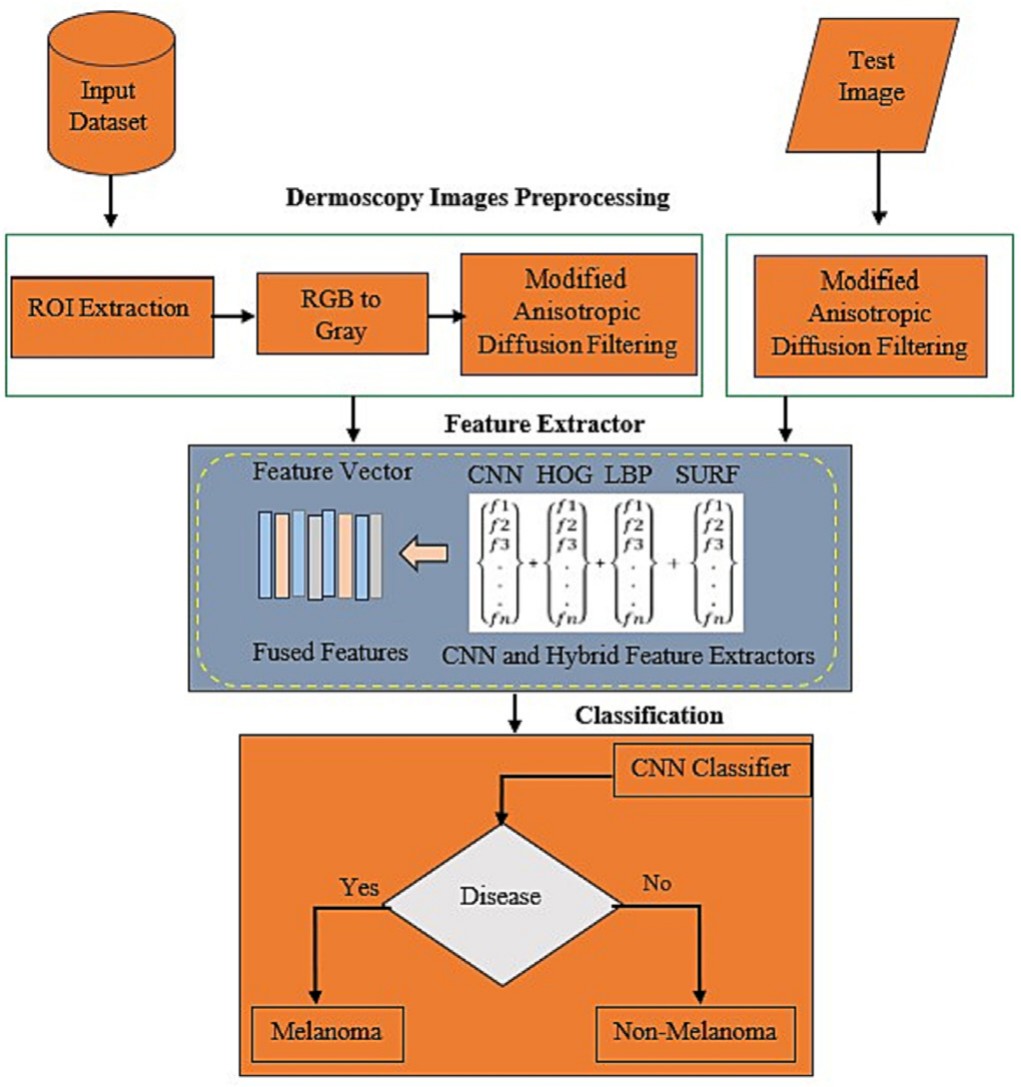
Diagram of our proposed system.

This section outlines our suggested strategy and the methods used for recognizing skin cancer. To remove the distracting and unwanted portions of the chosen Region of Interest (ROI), we first truncate the image and then convert it from RGB to grayscale. The prediction performance is improved by using a modified version of anisotropic diffusion filtering. The train and test images have multiplicative noise that is removed via the apostrophic diffusion filtering approach. In addition, we suggest 1 feature extractors: a Hybrid Feature Extractor (HFE) and a feature extractor based on a Convolutional Neural Network (CNN). The HFE creates a fused feature vector by fusing together 3 feature extractors: HOG, LBP, and SURF. The suggested technique uses an entropy-based feature selection mechanism to choose the best features from non-optimized fused features. The CNN is then used to classify the skin cancer as melanoma or non-melanoma. Fig. 1, shows the general framework of our proposed methodology.

This section also offers a novel approach for feature extraction and categorization known as Algorithm 1. The entire process is broken down into several sections. First, a dermatological image of skin cancer is used as input during the input stage for the train and test data. Features are divided into 2 distinct parts during the extraction stage. One of them uses an extraction based on CNN, while the other uses a hybrid extraction. The maximum number of attributes in CNN that can be preserved in F_C_ is 1570. The distinct characteristics of L_0_, L_1_, and L_2_ are combined into a fused vector known as HFE in the hybrid section. All features are ultimately merged into a vector called V throughout the extraction stage. Then melanoma and non-melanoma are classified. In the next subsections, the requirements for each step of the suggested scheme are presented.

Algorithm 1 Proposed algorithm of Melanoma skin cancer detection.

**Table:** 

### Dataset

**Fig. 2:**
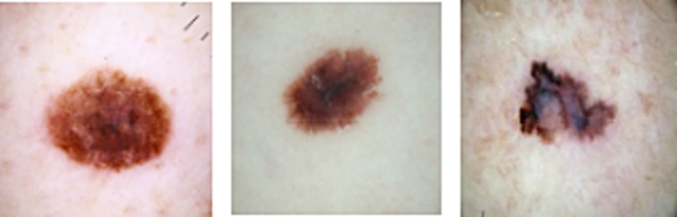
Sample image of skin cancer.

Over the course of 20 years, the Department of Dermatology at the Medical University of Vienna, Austria, and Cliff Rosendahl's skin cancer clinic in Queensland, Australia, contributed the dermatoscopic images contained in the HAM10000 training set. Dermatoscopic images from various populations that have been gathered and archived using various techniques. In this study, we use this variant and publicly available standard dataset from Academic torrents dataset.^39^ The dataset contains 16 170 RGB images in distinct types: melanoma and non-melanoma. All of the skin cancer images have a resolution of 1980×2022. The entire dataset is divided into training (70%) and testing (30%) sets. Fig. 2 shows some samples image of our taken dataset.

### Data preprocessing

**Fig. 3:**
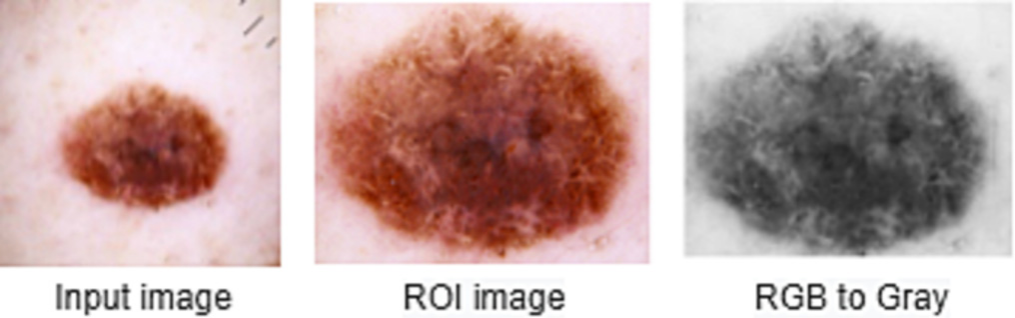
Image data processing.

Data preprocessing is the most essential task for analysis before applying any feature extraction and classification methods.^40^, ^41^, ^42^ Each image has a group of pixels that contains noise and imperfection. Several procedures are used to remove redundant pixels and distortion pixels from images in order to produce correct outcomes.^73^ After performing the ROI technique, the proposed system converts the RGB image to grayscale. In order to remove irrelevant text and machine annotations from training and test images, the Region Of Interest (ROI) is extracted.^43^ This approach suppresses the number of unnecessary noise and distortion. Fig. 3 demonstrates the procedure of image data preprocessing. The proposed system used a starburst pattern and poor dermatological images during the testing phase. A modified apostrophic diffusion filtering approach is used to eliminate multiplicative noise present in the test image. Hence, it is capable of efficiently overcoming the challenges of a noisy image. As a result, the preprocessing approach for the input text image is more successful in extracting reliable features.

For any supervised learning for training phase, it is necessary to collect a huge volume of labeled data. In most applications, inadequate training data might lead to an overfitting issue.^22^^,^^44^ By erasing the overfitting status, the data augmentation approach is able to overcome this barrier. The best fit in our model is a machine learning based on CNN model, which overcomes the limitation of the shortage of labeled images. Some of the augmentation techniques include translation, resizing, slicing, magnification, rotation, reversing, and brightness adjustment that may be used to change the size and appearance of a lesion in a dermatological image.

### Modified Anisotropic Diffusion Filtering (MADF)

The objective of proposed Modified Anisotropic Diffusion Filtering is to preserve information while speckles are being reduced. The suggested approach uses covariance and kurtosis measurements of noise to maintain the critical edge information. This speckle reduction technique is continued until the image’s noise component reaches to Gaussian value. If the distortion is Gaussian, the skewness value must be 0. Equation (1) represents the noise component. The loop will remain until the kurtosis of noise part is less than the measurement. This measurement can be defined by equation (3). When the relationship with both image class and disturbance class is the smallest, the iteration will end. In equations (1), (2), (3), (4), (5), (6), (7), I and I_0_ represent actual and noisy image, μ is used to represent the mean of noise intensity G. The kurtosis value k is determined by equation (4). Equation (6) derives the image intensity correlation, whereas Equation (7) generates the noise intensity correlation. When I and G have the lowest amount of deviation, the recommended filtering will produce reliable results.

(1) $$n=\frac{I-G}{\sqrt{G}}$$

(2) $$I_{0}=I_{n}$$

(3) $$\mu =\frac{\sum_{i=1}^{N}G_{i}}{N}$$

(4) $$k=\frac{\frac{1}{N}\sum_{i=0}^{N}{\left(G-\mu \right)}^{4}}{{\left[\frac{1}{N}\sum_{i=0}^{N}{\left(G-\mu \right)}^{2}\right]}^{2}}-3$$

(5) $$\mathrm{abs}\left(n-k\right)\leq 0.00$$

(6) $$\rho_{I}=\frac{\sum_{i=0}^{M-1}\sum_{j=0}^{N-1}i.j.p_{I}\left(i,j\right)-\mu I_{x}\mu I_{y}}{\frac{\sum_{i=1}^{N}\left(I_{\mathrm{ix}}-\mu_{\mathrm{Ix}}\right)(I_{\mathrm{iy}}-\mu_{\mathrm{Iy}})}{N}}$$

(7) $$\rho_{G}=\frac{\sum_{i=0}^{M-1}\sum_{j=0}^{N-1}i.j.p_{G}\left(i,j\right)-\mu G_{x}\mu G_{y}}{\frac{\sum_{i=1}^{N}\left(G_{\mathrm{ix}}-\mu_{\mathrm{Ix}}\right)(G_{\mathrm{iy}}-\mu_{\mathrm{Iy}})}{N}}$$

**Fig. 4:**
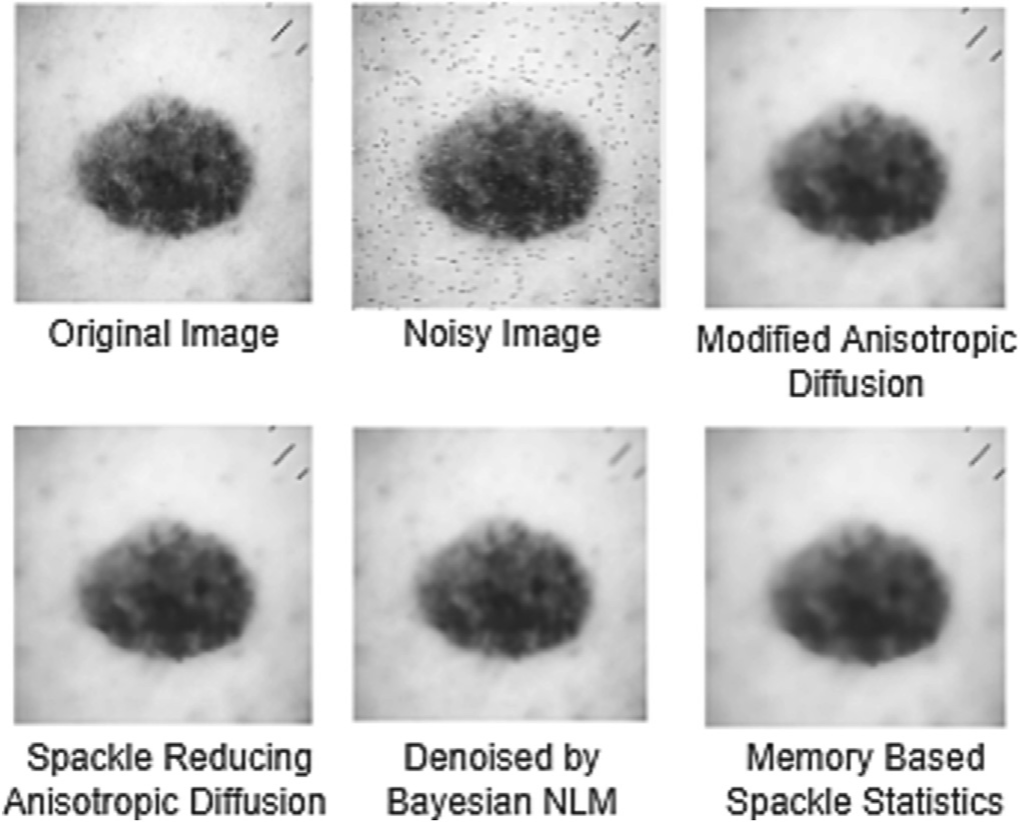
Comparison among different Anisotropic Diffusion techniques.

Fig 4 depicts a visual comparison of speckle-reducing anisotropic diffusion, Bayesian NLM-demised, Memory-based Speckle Statistics, and our recommended Modified Anisotropic Diffusion.

### Feature extraction

In this section, we present different feature extraction techniques used in this study. Here, we used 2 image extractors namely, HFE and a CNN. HOG, LBP, and SURF are 3 feature extraction techniques included in the HFE extractor. Numerous machines learning and computer vision applications use data fusion.^20^^,^^45^, ^46^, ^47^, ^48^, ^49^^,^^74^ Here, we propose a method based on entropy-based feature fusion. In this method, the entropy is applied to the features vector for the selection of optimal attributes based on the entropy score. Equations (8), (9) explain how the feature selection method works mathematically. From a total of 7948 characteristics, entropy was utilized to choose 1280 score-based characteristics. Equations (8), (9) indicate entropy and denote features probability. In order to classify melanoma skin cancer images, the final attributes are used for classification purpose.

(8) $$B_{\mathrm{He}}=-N{\mathrm{He}}_{b}\sum_{i=1}^{n}p\left(f\right)$$

(9) $$F_{\text{select}}=B_{\mathrm{He}}\left(\max\left(f_{i,}1280\right)\right)$$

**Fig. 5:**
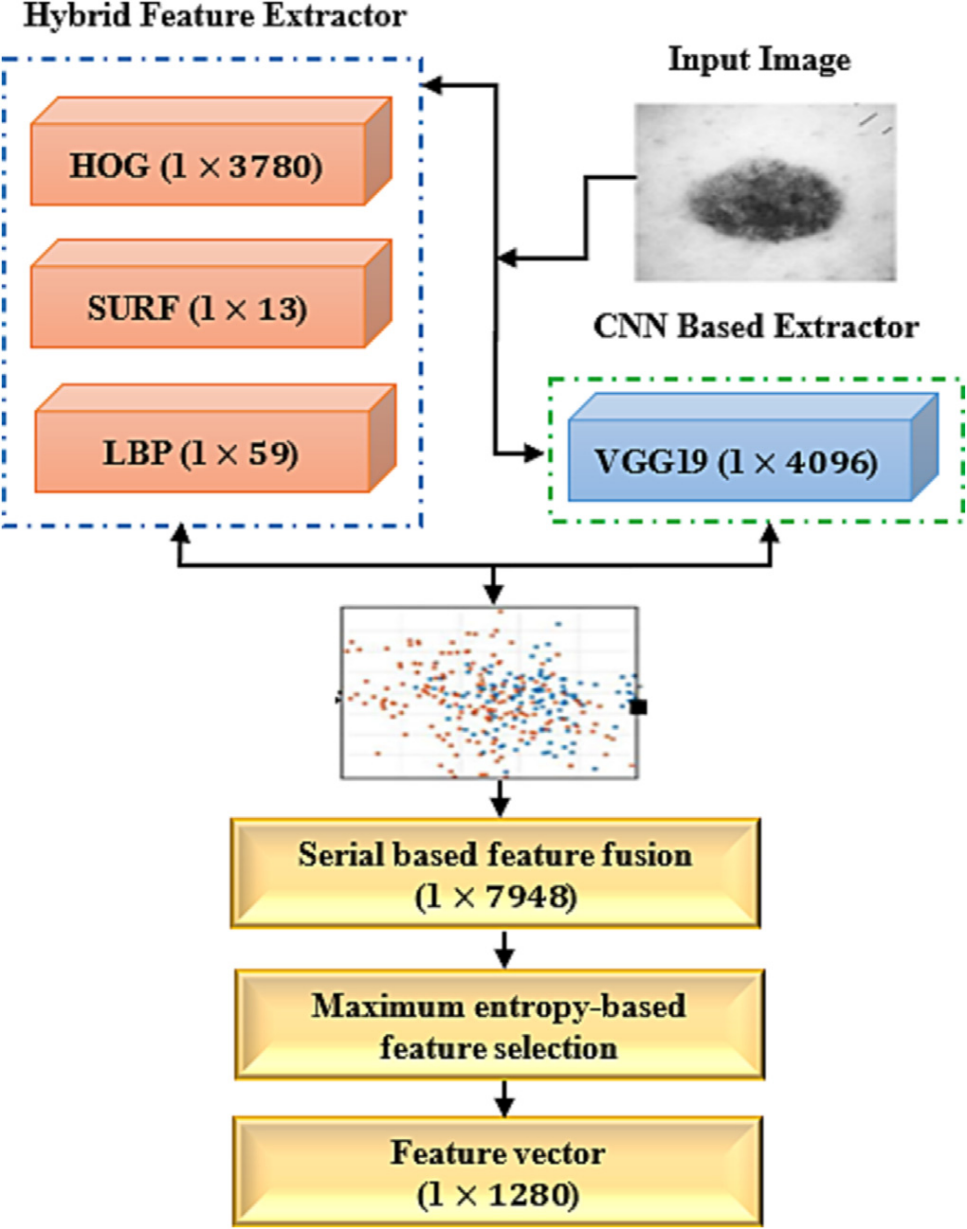
Combination of feature fusion extractor.

Fig. 5 depicts the segmentation and selection procedure. The proposed method has been tested on a fused features vector that includes both hybrid and deep learning features.

### Hybrid feature extractor

Three feature extraction strategies included in the HFE extractor are: Local Binary Pattern (LBP), Histogram-Oriented Gradient (HOG), and Speed Up Robust Feature (SURF), all of which result in a single fused feature vector (equations (10), (11), (12)). HOG properties are extracted from the images at all grid dense regions and are often utilized for object detection. HOG attributes (1×3780), LBP attributes (1×59), and SURF attributes (1×13). All features are used to define the form and appearance of skin cancer. These 3 feature extraction methods are explained in detail in the following sections.

(10) $$f_{\mathrm{HOG}\;1\mathrm{xn}}=\left\{{\mathrm{HOG}}_{1x1},{\mathrm{HOG}}_{1x2},{\mathrm{HOG}}_{1x3}\ldots {\mathrm{HOG}}_{1\mathrm{xn}}\right\}$$

(11) $$f_{\text{SURF}\;1\mathrm{xm}}=\left\{{\text{SURG}}_{1x1},{\text{SURF}}_{1x2},{\text{SURF}}_{1x3}\ldots {\text{SURF}}_{1\mathrm{xm}}\right\}$$

(12) $$f_{\mathrm{LBP}\;1\mathrm{xp}}=\left\{{\mathrm{LBP}}_{1x1},{\mathrm{LBP}}_{1x2},{\mathrm{LBP}}_{1x3}\ldots {\mathrm{LBP}}_{1\mathrm{xp}}\right\}$$

Furthermore, the extracted features are combined into 1 vector.

(13) $$\text{Fused}{\left(\text{features vector}\right)}_{1\mathrm{xq}}=\sum_{i=1}^{3}\left\{f_{\mathrm{HOG}\;1\mathrm{xn}},f_{\text{SURF}\;1\mathrm{xm}},f_{\mathrm{LBP}\;1\mathrm{xp}}\right\}$$

### Histogram-oriented gradient features

**Fig. 6:**
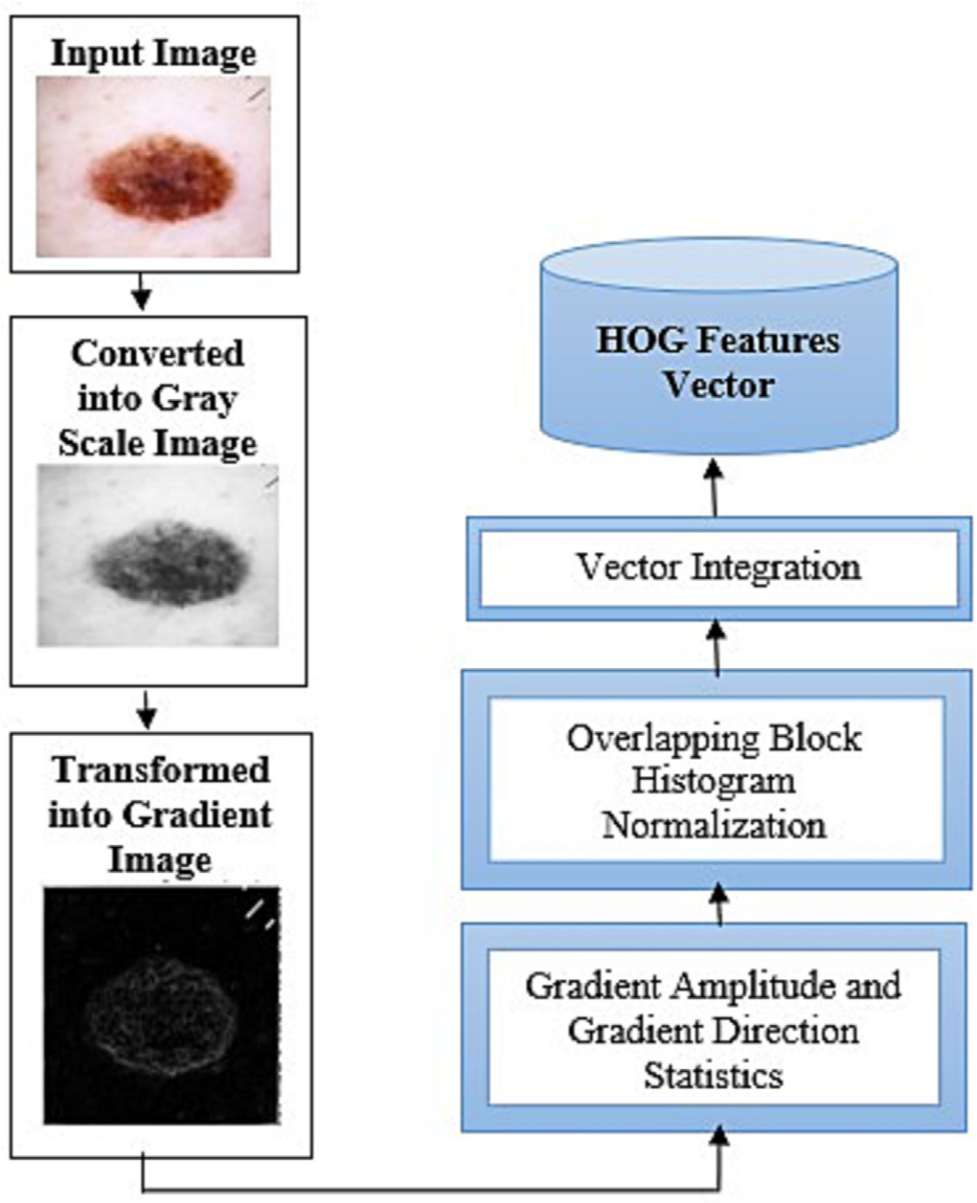
The HOG extracting features algorithm's basic process.

In this method, the input image is first converted into grayscale image. After that, images are transformed into gradient image for better edge detection. The gradients or edges orientation histogram is obtained in each cell unit, divided into smaller cells, and then these histograms are combined to give a HOG description.^50^^,^^51^ Fig. 6 depicts the HOG feature extraction algorithm’s fundamental flow.

To extract the feature in HOG, we should first create a gradient on both the x and y axis. x and y direction slopes can be determined fast since the horizontal direction's pattern is K=[-1, 0, 1], and its inversion may be used to filter an image. The following is the indication:

(14) $$g_{x}=I\left(x+1,y\right)-I\left(x-1,y\right)$$

(15) $$g_{y}=I\left(x,y+1\right)-I\left(x,y-1\right)$$

The pixel value of (x, y) is indicated by I, and the direction slope of x is represented by g_x_, and the orientation slope of y is indicated by g_y_. The slope magnitude of (x, y) is represented by g (x, y) and denoted by

(16) $$∆g\left(x,y\right)=\sqrt{\left(g_{x}^{2}+g_{y}^{2}\right)}$$

And (x, y) gradient 's direction (θ) is determined as follows:

(17) θ=arctangygx

### Local Binary Pattern (LBP) features

LBP provides texture analysis and local spatial statistics of ultrasound image.^52^ A threshold value is used to level the contiguous pixels and it is represented by 0 and 1. If each pixel value is larger than the center pixel value, each adjacent pixel gray value (3×3) is leveled as 1, otherwise it is leveled as 0. Thus, LBP represents a set of binary digits which are used to replace center pixel value after converting into decimal. Equations (18), (19) represent LBP segmentation from test image where g (p) is gray level pixel for surrounding pixels (i, j) and g (c) is complementary constant. For neighbor (8, i), the total number of samples is 256.

(18) $$l=\sum_{P=0}^{P-1}S\left(g_{p}-g_{c}\right)2^{P}$$

(19) S=1;iflij>00;otherwise

### Speed Up Robust Feature (SURF) features

SURF is a similarity invariant representation and comparison algorithm. Its robust feature extractor technique is used in nearest neighbor matching.^53^ During augmentation, it can extract features. As a scaling and rotation variant algorithm, SURF provides fast operator computation using box filtering.^54^ The 2 functions of SURF are feature extraction and feature description. The features extraction in SURF is done with Hessian matrix-based interest point approximation. The SURF descriptor provides unique information of features generated by surrounding area of an interest point. It operates by indicating the distinctive orientation of an interesting point using Haar wavelet responses. Before calculating descriptor, interest areas of neighbor interest point are rotated to its selected orientation. The Hessian matrix H (x, y) at scaling is given by formula (20) for a given location X = (x, y). Equation (21) represents the wavelet response in x and y direction is noted by dx and dy direction. A vector V is computed for each sub region.

(20) $$H\left(\dot{x},\sigma \right)\left[\begin{array}{cc} L_{\mathrm{xx}}\left(x,\sigma \right) & L_{\mathrm{xy}}\left(x,\sigma \right)\\ L_{\mathrm{xy}}\left(x,\sigma \right) & L_{\mathrm{yy}}\left(x,\sigma \right) \end{array}\right]$$

(21) $$V=\left\{\sum \mathrm{dx},\sum \mathrm{dy},\sum |\mathrm{dx}|,\sum \left|\mathrm{dy}\right|\right\}$$

### CNN-based feature extraction and classification

**Fig. 7:**
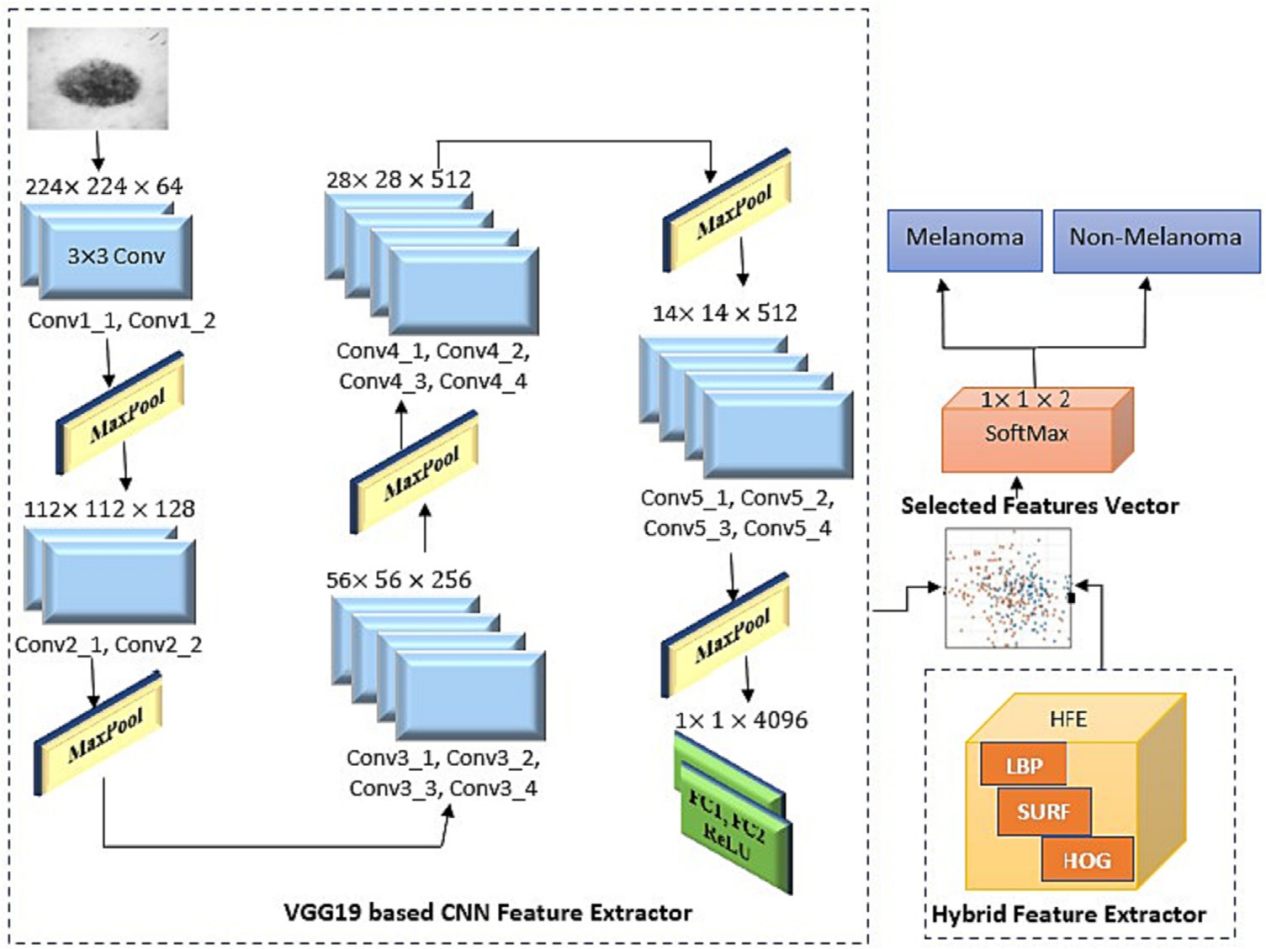
CNN- and HFE-based proposed architecture.

CNN-based feature extraction techniques are most popular process in medical image processing.^55^ Multi-tasking cascaded CNN is the most used features extraction method in classification.^56^ Here, we studied pre-trained VGGNet, VGG16, Scratch model, ResNet50, and AlexNet which among them, VGG19 attained the best results. VGG19 consists of 19 layers. Fig. 7 shows how VGG19 was constructed using 16 convolution layers and 3 completely linked layers.

The convolution component is separated into 5 successive max-pooling layers, with a nonlinear ReLU function acting as an activation function to ensure that each convolution layer’s output is more accurate. Depth of the 5 consecutive layers are 64, 128, 256, 512, and 512, respectively. Each of the layers formatted with sub regions where pooling layer decrease the learnable parameter. The final layer played a vital role to get feature vector of proposed VGG19 model. Every fully attached layer besides the dropout layers are regularized using L2 to reduce overfitting impact during implementation of the fine-tuned model. Applying ReLU function on VGG19-based CNN model produce 4096 tuned features. The features of VGG19 based CNN model and Hybrid feature extractor features are then fused to build a single feature vector. The graphical illustration of feature extraction step is shown in Fig. 7. The computational complexity of the proposed approach is of O (n).

### Finding a cancer region

**Fig. 8:**
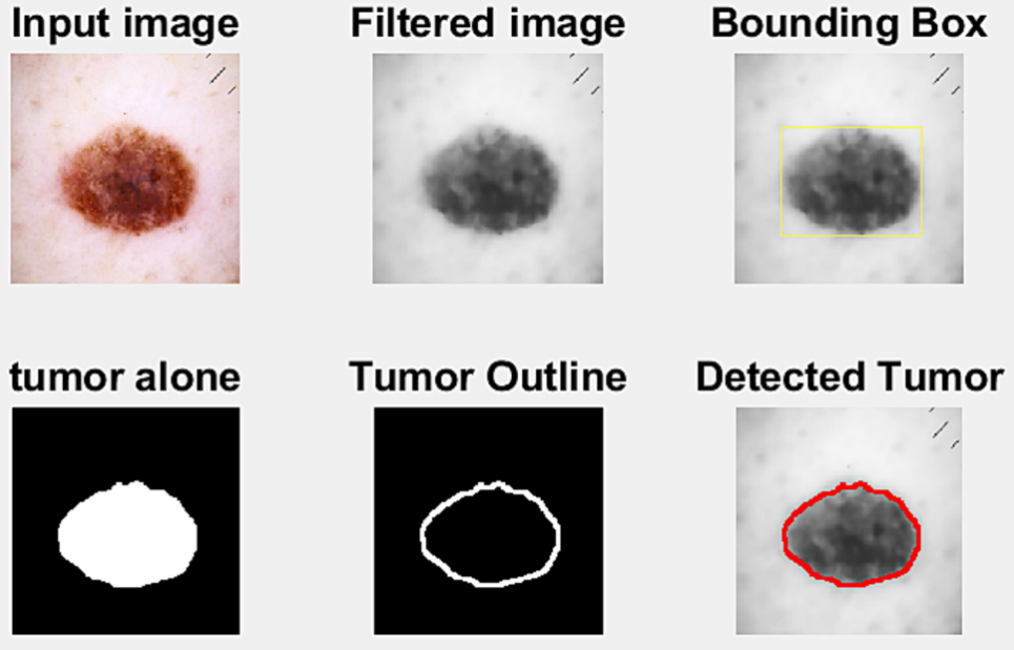
Detect fracture region from skin cancer.

Depending on the nature of the lesion, skin lesions appear in a range of forms and sizes. A circle is drawn around any spot that has been identified as a lesion. Fig. 8 shows the process of divulging tumor from skin lesion images. As it was discussed earlier, dermoscopic skin lesion images are taken in training phase. Input images are transformed from RGB to grayscale prior to preprocessing. A modified anisotropic diffusion filtering method is used to the intended pictures to remove undesired noises. As a result, filtered images provide an accurate region of tumor when fast bounding box is applied on it.

Sometimes, variant segmentation techniques are used to tumor detection which are not convenient and efficient due to deformation growth of lesion. Finding accurate region of skin lesion and symmetry of axis is time-consuming and challenging. Researchers nowadays uses fast bounding box to detect lesion faster and robust. A fast-bound box technique is fast and robust process of segmentation which overcome the above problem by locating an axis parallel box or bounding box around the skin lesion. This process is scored based on grayscale intensity analysis. Score function provides a linear search method for bounding boxes. FBB is an unsupervised and real-time basis process where images fixation is not mandatory. Using this boundary box symmetry method tumor region can be detected alone finally which provides an outlier around the tumor.

## Experimental setup

### Environmental setup

Matlab R2020a was used to develop the proposed feature fusion architecture on a Windows 11 64-bit PC. The machine has 8 GB of RAM and a 2.80 GHz Intel 11th Generation Core i7-1165G7 processor.

### Confusion matrix

A confusion matrix is a form of data that determines a classifier's prediction performance. The number of right and wrong predictions is summed using this approach, and it is seen which machine learning classifiers work effectively. The classifiers employed in this study have been tested and compared in this section. Table 2 shows the performance of each classifier. In this study, we use 4 evaluation metrics namely, accuracy, sensitivity, specificity, and precision. The parameters used to compute each measure are True Positive (TP), False Positive (FP), True Negative (TN), and False Negative (FN). In Table 2, confusion metrics are derived by calculating different values of TP, TN, FP, and FN. Equations (22), (23), (24), (25) represent the computing formula of these performance metrics.

(22) $$\text{Accuracy}\;\left(\mathrm{ACC}\right)=\frac{\mathrm{TP}+\mathrm{TN}}{\mathrm{TP}+\mathrm{TN}+\mathrm{FP}+\mathrm{FN}}$$

(23) $$\text{Sensitivity}\;\left(\mathrm{SEN}\right)=\frac{\mathrm{TP}}{\mathrm{TP}+\mathrm{FN}}$$

(24) $$\text{Specificity}\;\left(\text{SPEC}\right)=\frac{\mathrm{TN}}{\mathrm{TN}+\mathrm{FP}}$$

(25) $$\text{Precision}\;\left(\text{PREC}\right)=\frac{\mathrm{TP}}{\mathrm{TP}+\mathrm{FP}}$$

**Table 2:** Confusion metrics of dataset.

True label	Melanoma	Non-melanoma
Melanoma	TP (11076)	FN (120)
Non-melanoma	FP (94)	TN (4880)

In Table 2, confusion metrics are derived by calculating different values of TP, TN, FP, and FN.

## Results and discussion

Dermatology skin images with various speckles, noises, and resolutions were employed as test data for the proposed approach. Information reserve and noise reduction are required when working with relevant features. To lessen the noise effects or data shortages, less restricted and heavier γ-quasi-cliques are considered in recent research.^57^ Imbalance data may be exploited productively by employing techniques such as preprocessing, algorithm modification, and feature selection.^58^ Several approaches are employed in this study to eliminate unnecessary pixels and distortion pixels from images in order to generate proper results. The suggested method transforms the RGB image to grayscale after executing the ROI approach. Then, before using the feature selection, a modified filtering method known as Modified Apostrophic Diffusion Filtering is applied on test and train dataset. In the classification process, VGG19-based CNN classifiers are used to overcome imbalance problems like overfitting.

In the image preprocessing stage, the desired system employed modified anisotropic diffusion filtering. Three evaluation metrics are used to quantify performance in modified anisotropic diffusion filtering namely, Edge Preservation Factors (EPF), Minimum Square Error (MSE), and Signal to Noise Ratio (SNR). Higher SNR and EPF values indicate superior noise removal and edge detail preservation, respectively.^72^ On the other hand, minimum MSE value shows that there is less inconsistency between the input and filtering images. Table 3 represents the performance measurement. Although all known filters perform well with respect to MSE, the proposed modified anisotropic filtering approach has a higher SNR and EPF.

**Table 3:** Anisotropic diffusion-based filtering algorithms to assess performance.

Methods	Evaluation criteria		
	SNR	EPF	MSE
SRAD	31.3683	0.7218	0.6648
OBLMN	30.2361	0.7147	0.6750
ADMSS	33.0854	0.7298	0.6846
**Proposed MADF**	**36.9687**	**0.9522**	**0.7389**

CNN-based models like VGG-16, AlexNet, VGG-19, and ResNet50 are widely used. All of these models provide the same result when given a similar training dataset. Table 4 represents performance of these different CNN-based models for experimental data. Trained feature calculations can be used to improve forecast success or failure.^59^^,^^74^^,^^75^ We also use CNN classifier as classification scheme. As shown in Table 4, the fine-tuned VGG19 pretrained model provides better result compared to other models. We may create a model that is efficient, focused, and effective by correctly using specificity and basing it on gains and goals. ResNet50 and VGG19 outperform other models in terms of specificity, as seen in Table 4. ResNet50, VGG16, and VGG19 pretrained models perform significantly better than Scratch and AlexNet models on the basis of accuracy. But considering all the performance parameters, VGG19 was selected to build our model.

**Table 4:** Performance measurement of different CNN models.

CNN architecture	Accuracy	Specificity	Sensitivity
Scratch model	0.8637	0.8345	0.8192
AlexNet	0.8727	0.8528	0.8343
ResNet50	0.9534	0.9345	0.9123
VGG-16	0.9153	0.8732	0.8923
**VGG19**	**0.9849**	**0.9460**	**0.9165**

Table 5 depicts a comparison of several feature extraction methods. As shown in this table, fusion increases the accuracy by integrating poor estimating models to produce a strong predicting model.^60^, ^61^, ^62^ When we apply traditional machine learning models like LBP, SURF, and HOG, we get accuracy less than 95%. But if we apply those 3 models to a feature extractor (HFE), it provides 97% accuracy, approximately. Individually, VGG19 provides 96% accuracy. When we combined VGG19 and the feature extractor, the accuracy reached 99%. Using fusion of all the extracted features, we achieve better results than using each of them, individually.

**Table 5:** Comparative result of feature fusion.

Proposed methods	Accuracy	Specificity	Sensitivity
VGG19	0.9673	0.8752	0.8673
LBP	0.9173	0.9253	0.9173
SURF	0.9271	0.9473	0.9356
HOG	0.9073	0.9376	0.9273
HFE (LBP+SURF+HOG)	0.9776	0.9673	0.9487
**Proposed method (VGG19+HFE)**	**0.9985**	**0.9570**	**0.9165**

Table 6 shows accuracy, sensitivity, and specificity result of noisy and speckle test dataset. As shown in this table, without preprocessing the performance result of proposed fused features and CNN is not satisfactory.

**Table 6:** Results of test data segmentation without preprocessing.

Methods	Accuracy	Specificity	Sensitivity
CNN only	0.9273	0.8552	0.8773
**Proposed method (VGG19+HFE)**	**0.9775**	**0.9250**	**0.9100**

Aside from using CNN, we also compare the performance of several traditional machine learning models, such as Decision Tree (DT), Random Forest (RM), Artificial Neural Network (ANN), Support Vector Machine (SVM), and K-Nearest Neighbor (KNN). The result of this comparison is presented in Table 7. As shown in this table, CNN achieves significantly better results which demonstrates the effectiveness of using deep learning model compared to traditional machine learning models.

**Table 7:** Comparative results of different classifier.

Classifier models	Accuracy	Specificity	Sensitivity
DT	0.8128	0.8023	0.7978
RF	0.8326	0.8293	0.8027
ANN	0.8756	0.8634	0.8523
KNN	0.9123	0.8823	0.8732
SVM	0.9532	0.9423	0.9343
**CNN**	**0.9985**	**0.9870**	**0.9765**

To obtain a more detailed experimental outcome, a 5-fold cross-validation procedure is also used to evaluate our model. In this model, the data is divided into 5 groups. In each step, 1 group is used for testing purpose and the remaining 4 are used for training. This process is repeated 5 times and until all 5 groups is used exactly once for testing purpose. Table 8 shows the results of 5-fold cross-validation when using features extracted by HFE, CNN, and combination of both. As shown in this table, the results achieved for 5-fold cross-validation is consistent with the results on the test set which demonstrates the generality of our proposed approach.

**Table 8:** 5-fold cross validation was used to evaluate overall classification accuracy.

Feature extraction techniques	Fold 1	Fold 2	Fold 3	Fold 4	Fold 5	Mean accuracy
HFE	0.8732	0.8789	0.8741	0.8675	0.8730	0.8734
CNN	0.9378	0.9367	0.9387	0.9367	0.9321	0.9364
**Proposed method (HFE+CNN)**	**0.9856**	**0.9887**	**0.9913**	**0.9797**	**0.9973**	**0.9885**

The result achieved for our proposed model compared to the state-of-the-art approaches for skin cancer detection is presented in Table 9. As shown in this table, we achieve 99.85% prediction accuracy which is 4.99% better than the best result reported in previous studies.^20^ This result demonstrates the performance of our model compared to those presented in previous studies.

**Table 9:** Existing melanoma detection algorithms are compared.

Authors	Dataset	Methodology (Feature extractor, classifier)	Accuracy (%)
Yuexiang Li et al.^14^	2000 images	Fully convolutional residual networks (FCRN), CNN	91.2
Vijayalakshmi M et al.^15^	1000–1500 images	CNN, SVM	85
A. Pramanik and R. Chakraborty^16^	100 images	CNN, CNN	87.58
Andre Esteva et al.^17^	129 450 images	CNN, CNN	72.1
K. Jayapriya et al.^18^		CNN, FCRN	88.92
Haenssle et al.^19^	300 images	Deep CNN, Google Inception V4	86.6
Dorj et al.^20^	3753images	ECOC SVM, CNN	95.1
**Proposed method**	**16170 dermatology images**	**(HFE+CNN), CNN**	**99.85**

## Conclusion

Today, it is important to use innovative techniques to identify medical biomarkers and classify diseases including cancer.^34^^,^^37^^,^^63^, ^64^, ^65^, ^66^, ^67^, ^68^, ^69^, ^70^, ^71^ Here, we proposed a machine learning model using hybrid feature extraction and convolutional neural network to detect skin cancer from dermatology images. We also used a modified anisotropic filtering technique in dermatology image to diverge speckle from noisy images. The proposed fused vector with CNN and HFE achieves 99.85% prediction accuracy. The best performing CNN classifier used to detect whether it’s melanoma or non-melanoma skin cancer achieves 99.85% prediction accuracy which is 4.99% better than those results reported in the literature. Such results demonstrate the preference of our proposed model compared to those models presented in previous studies. The fused approach outperforms the conventional method in terms of performance and accuracy. One of the shortcomings of our proposed method is that it is computationally demanding. Running the program with fusion features takes a long time. Our future direction is to reduce the number of extracted features and use faster classification models to reduce the computational time of skin cancer detection.

## Authorship contribution

MMR designed the study. MMR and MNA wrote the manuscript; MMR, and MNA collected data. MMR, MSIK, MNA, MNA edited the manuscript; MMR, MKN, and MSIK carried out the analyses. MMR, and MNA generated all figures and tables.

## Conflict of interest

The authors declare that they have no competing interests.

## Data Availability

All the materials can be retrieved from https://github.com/shimulmbstu/skin-cancer-codes.
